# Comparative analysis of bats and rodents’ genomes suggests a relation between non-LTR retrotransposons, cancer incidence, and ageing

**DOI:** 10.1038/s41598-023-36006-6

**Published:** 2023-06-03

**Authors:** Marco Ricci, Valentina Peona, Alessio Boattini, Cristian Taccioli

**Affiliations:** 1 Independent Researcher, Gatteo, Italy; 2 Department of Organismal Biology, Systematic Biology, Uppsala University, Uppsala, Sweden; 3 Department of Biological, Geological and Environmental Sciences, University of Bologna, Bologna, Italy; 4 Department of Animal Medicine, Health and Production, University of Padova, Padua, Italy

**Keywords:** Cancer, Zoology, Interspersed repetitive sequences, Genetics, Mobile elements

## Abstract

The presence in nature of species showing drastic differences in lifespan and cancer incidence has recently increased the interest of the scientific community. In particular, the adaptations and the genomic features underlying the evolution of cancer-resistant and long-lived organisms have recently focused on transposable elements (TEs). In this study, we compared the content and dynamics of TE activity in the genomes of four rodent and six bat species exhibiting different lifespans and cancer susceptibility. Mouse, rat, and guinea pig genomes (short-lived and cancer-prone organisms) were compared with that of naked mole rat (*Heterocephalus glaber*) which is a cancer-resistant organism and the rodent with the longest lifespan. The long-lived bats of the genera *Myotis*, *Rhinolophus*, *Pteropus* and *Rousettus* were instead compared with *Molossus molossus*, which is one of the organisms with the shortest lifespan among the order Chiroptera. Despite previous hypotheses stating a substantial tolerance of TEs in bats, we found that long-lived bats and the naked mole rat share a marked decrease of non-LTR retrotransposons (LINEs and SINEs) accumulation in recent evolutionary times.

## Introduction

Transposable elements (TEs) are repetitive mobile elements present in almost all eukaryotes^1,2^. Many studies have shown that TEs are implicated in gene duplications, inversions, exon shuffling, gene expression regulation and may also play a role in the long-term evolution of eukaryotes^3–8^. For example, the co-option of TE-related proteins gave rise to vertebrate acquired immune system and mammalian placenta^9,10^. Although some TE insertions have been co-opted by genomes, the overall TE activity can be disruptive especially at short timescale. Indeed, TEs can be responsible for several human diseases^11–13^ like immunodeficiency^14^, coagulation defects^15^, cardiomyopathies^16^, and muscular dystrophies^17,18^. Interestingly, the dysregulation of TEs in somatic cells can lead to the establishment, and development of cancer^19–22^. The dysregulation of TE activity in cancer cells is so pervasive and accentuated that the methylation level of transposable elements is used as a biomarker for the malignancy of several types of tumours^23^. Given the manifold effects of TEs on health, it is reasonable to consider TE activity as a key factor able to influence the lifespan of several species^24^. In this study, we investigate the possible association between TE activity and lifespan by comparing the presence of TEs in genomes of mammals (rodents and bats) with different lifespans and cancer incidences. The TEs that cause mutations and genomic instability in the genomes are the ones currently active and able to move throughout the genome^24,25^. Since there are no transposition assays available for many organisms, we used the genetic divergence of the TE insertions (see “Methods” section) from their consensus sequences as a proxy for their active or inactive state^4^. Among all the mammalian species for which genome assemblies are publicly available, we chose four species of Rodentia and six of Chiroptera that: (1) have a high-quality genome assembly based on PacBio or Nanopore or Sanger sequencing data (in order to maximize the quality and quantity of transposable elements assembled^1^); (2) belong to the same taxonomical order (to maximize their shared evolutionary history); and (3) have comparable body masses. In particular, the inclusion of body mass among the criteria of selection allowed us to work with species that have not evolved biological adaptations to counteract cancer incidence as a function of body size (Peto’s paradox)^26^. In fact, animals with a large body mass evolved mechanisms to contrast the development of tumours such as the control of the telomerase expression^27^, or the expansion in copy number of coding genes and miRNAs^28,29^. Small mammals have a smaller number of cells and generally shorter life cycles^30^, therefore they are disentangled from the Peto’s paradox. For this reason, we selected mammals with a body mass lower than 2 kg to investigate the relationship between lifespan and TEs avoiding biases related to adaptations to large body masses. Thus, we tested the hypothesis that the genomes of cancer-prone and short-lived species present a higher load of recently inserted TEs compared to cancer-resistant and long-lived species.

## Results

**Figure 1:**
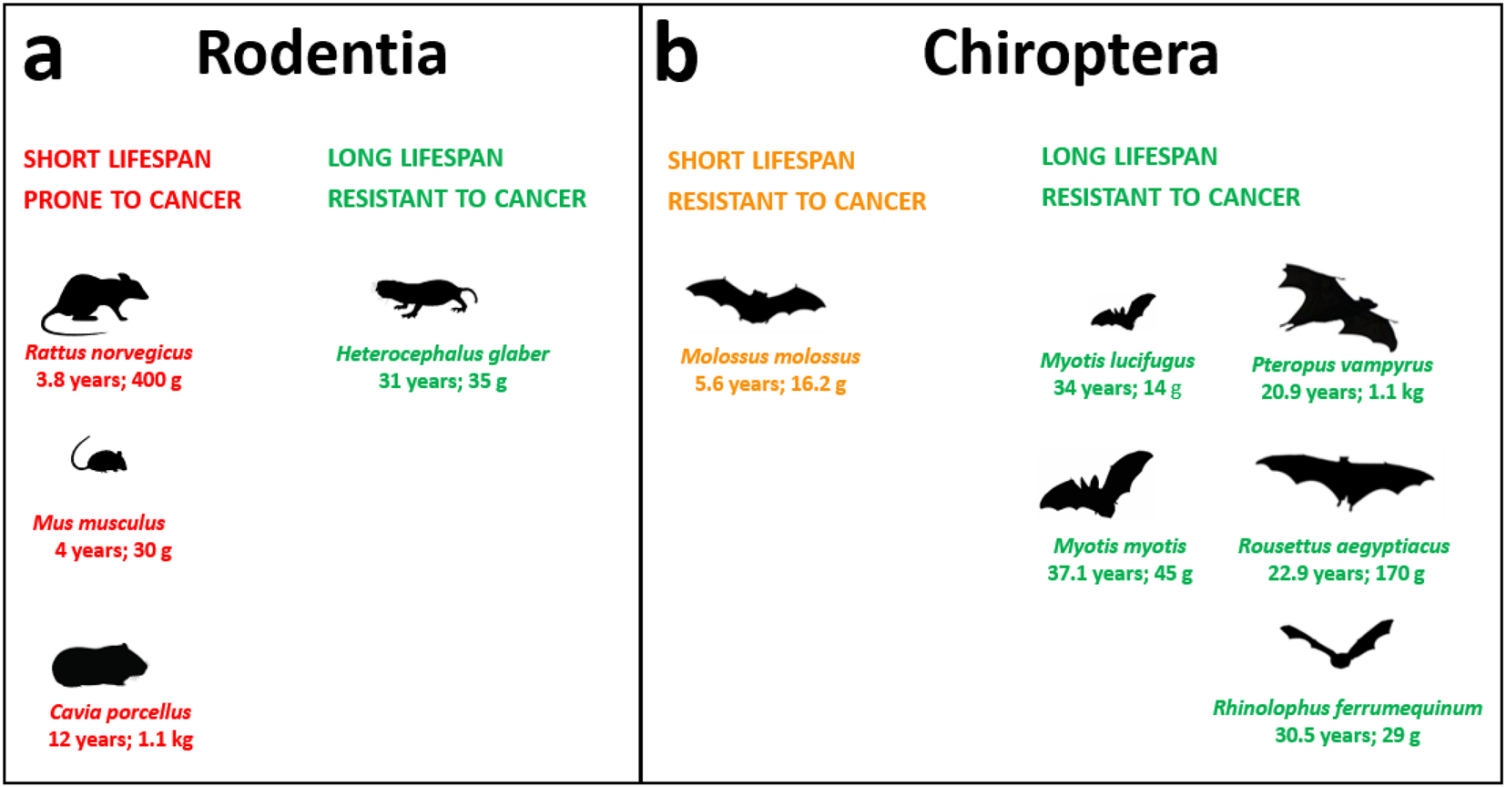
List of species analysed together with lifespan and body mass information. (**a**) Rodents with short lifespan and high cancer incidence are indicated in red while species with long lifespan and low cancer incidence are indicated in green. (**b**) Bats with short lifespan and low cancer incidence are indicated in orange while species with long lifespan and low cancer incidence are indicated in green. This information was retrieved from https://genomics.senescence.info/species/index.html and Speakman et al.^31^.

In this study we investigated the possible link between TEs, cancer incidence, and ageing by focusing on the accumulation patterns of TEs in genomes belonging to species showing different lifespans and cancer incidence (Fig. 1).

**Figure 2:**
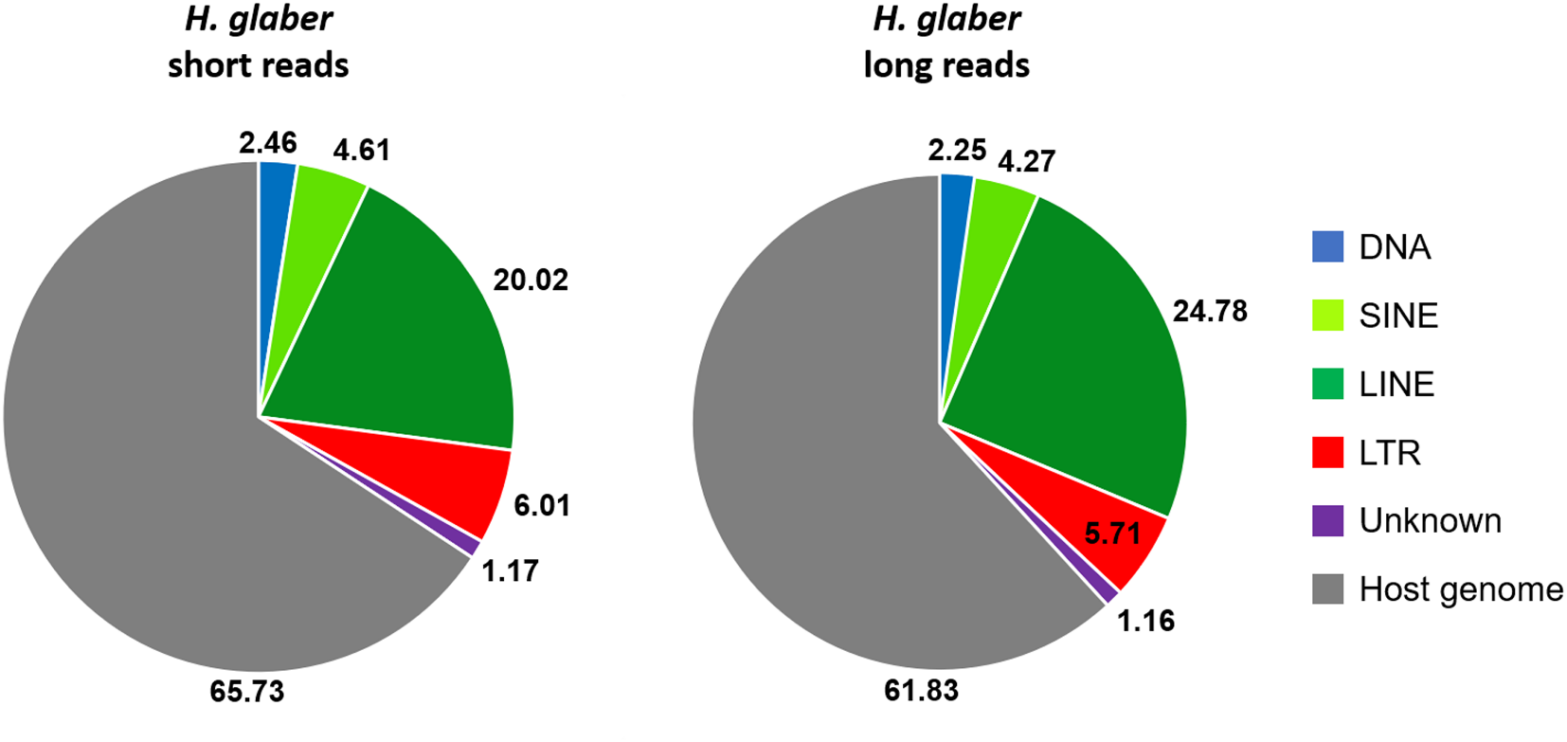
Transposable element content comparison between the two genome assemblies of *H. glaber*. The pie charts show the percentage of the main transposable element categories. The portion of the genome in grey comprises all the repetitive regions not annotated as transposable elements (e.g., tandem repeats and multi-copy gene families) as well as non-repetitive sequences. *H. glaber* short reads: HetGal_1.0, assembly size 2.6 Gb; *H. glaber* long reads (PacBio): Heter_glaber.v1.7_hic_pac, assembly size 3 Gb.

We collected genome assemblies of similar high quality and generated a TE library (Data S1) for the species that previously lacked one to both maximise the presence of TEs in the assemblies and their correct annotation (Tables 1, 2**)**. The de novo TE characterisation highlighted a higher percentage of TEs in the last genome assembly version of *H. glaber* based on PacBio technology compared to its first version based on short reads (Fig. 2).

**Table 1:** The assembly size and the percentage of genome annotated as transposable elements are shown for each rodent genome.

Species	*H. glaber*	*C. porcellus*	*M. musculus*	*R. norvegicus*
Assembly size (Gb)	3.042	2.723	2.728	2.648
Total TE content (%)	38.16	36.01	41.86	41.76
DNA transposons (%)	2.3	1.7	1.1	1.1
SINE (%)	4.3	5.5	7.6	7.2
LINE (%)	24.78	21.4	19.2	20.9
LTR (%)	5.7	7.4	12.6	10.7
Unknown (%)	1.2	1.7	1.4	1.9

**Table 2:** The assembly size and the percentage of genome annotated as transposable elements are shown for each bat genome.

Species	*M. myotis*	*M. lucifugus*	*R. ferrumequinum*	*R. aegyptiacus*	*P. vampyrus*	*M. molossus*
Assembly size (Gb)	2.003	2.035	2.075	1.893	1.996	2.319
Total TE content (%)	32.33	31.66	34.29	29.08	28.84	40.6
DNA transposons (%)	4.96	5.12	5.32	3.78	3.81	3.2
Helitrons (%)	6.93	6.18	0.06	0.06	0.06	0.52
SINE (%)	5.79	5.88	2.31	1.5	1.56	9.6
LINE (%)	15.62	14.76	20.13	17.74	17.33	22.6
LTR (%)	5.79	5.72	6.42	5.97	6.06	5.1
Unknown (%)	0.17	0.19	0.1	0.08	0.08	0.1

**Figure 3:**
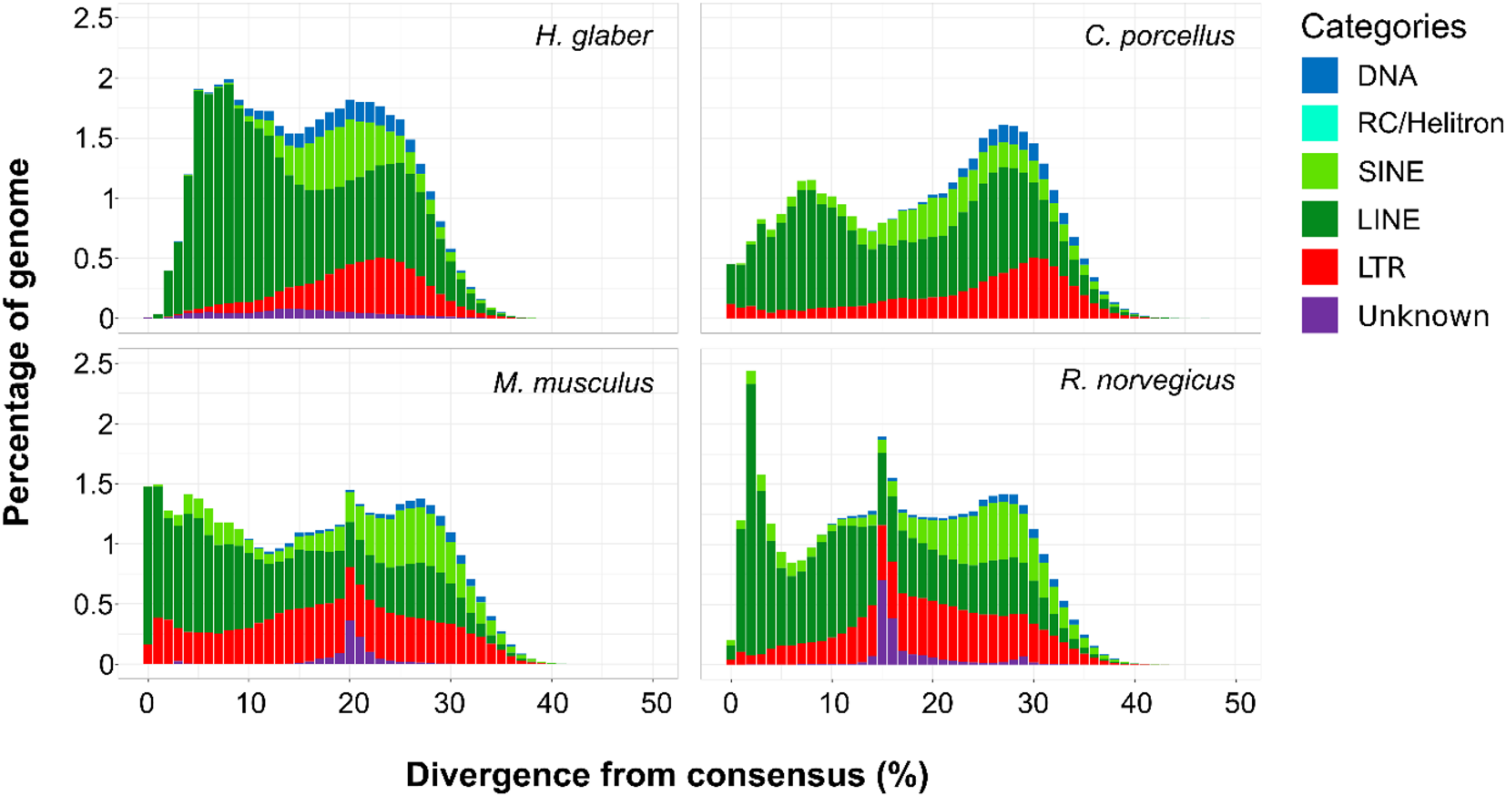
Transposable element landscapes of rodent species. The X-axis shows the genetic distance between the transposable element insertions and their consensus sequences (Kimura 2-p), whereas the Y-axis shows the percentage of genome annotated as transposable elements.

Then, we proceeded to compare the accumulation profiles of TEs between long- (*H. glaber*) and short-lived rodents (*M. musculus*, *R. norvegicus*, *C. porcellus*) using the output of RepeatMasker (Data S2–S11). We observed a reduced accumulation of transposable elements in the naked mole rat (*H. glaber*) in recent times with respect to the short-lived rodents (Fig. 3).

**Figure 4:**
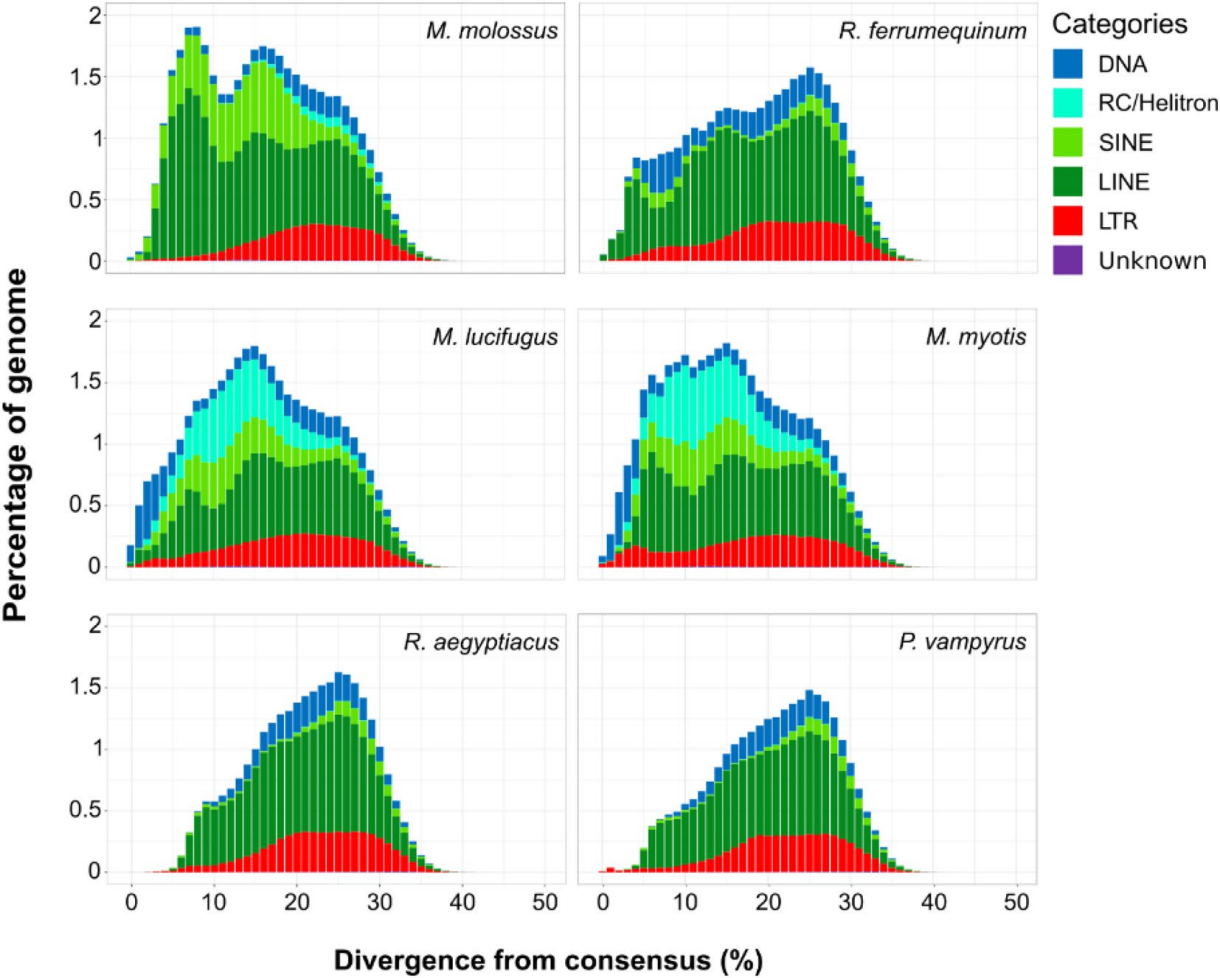
Transposable element landscapes of bat species. The X-axis shows the genetic distance between the transposable element insertions and their consensus sequences (Kimura 2-p), whereas the Y-axis shows the percentage of the genome annotated as transposable elements.

The same analysis was performed in bats. Bat genomes show a high accumulation of class II transposons in all species (Table 2) as well as a drop in the accumulation of non-LTR retrotransposons in recent times (Fig. 4).

The genomes of the analysed species are mainly composed of retrotransposons (SINEs, LINEs, LTRs). The main difference between the two orders is that all six bat genomes show a higher percentage of class II transposons (DNA transposons and Helitrons) compared to rodents, with a minimum percentage present in *M*. *molossus* (3.72%) and a maximum percentage in *M*. *myotis* and *M*. *lucifugus* (> 11%; Table 2).

**Figure 5:**
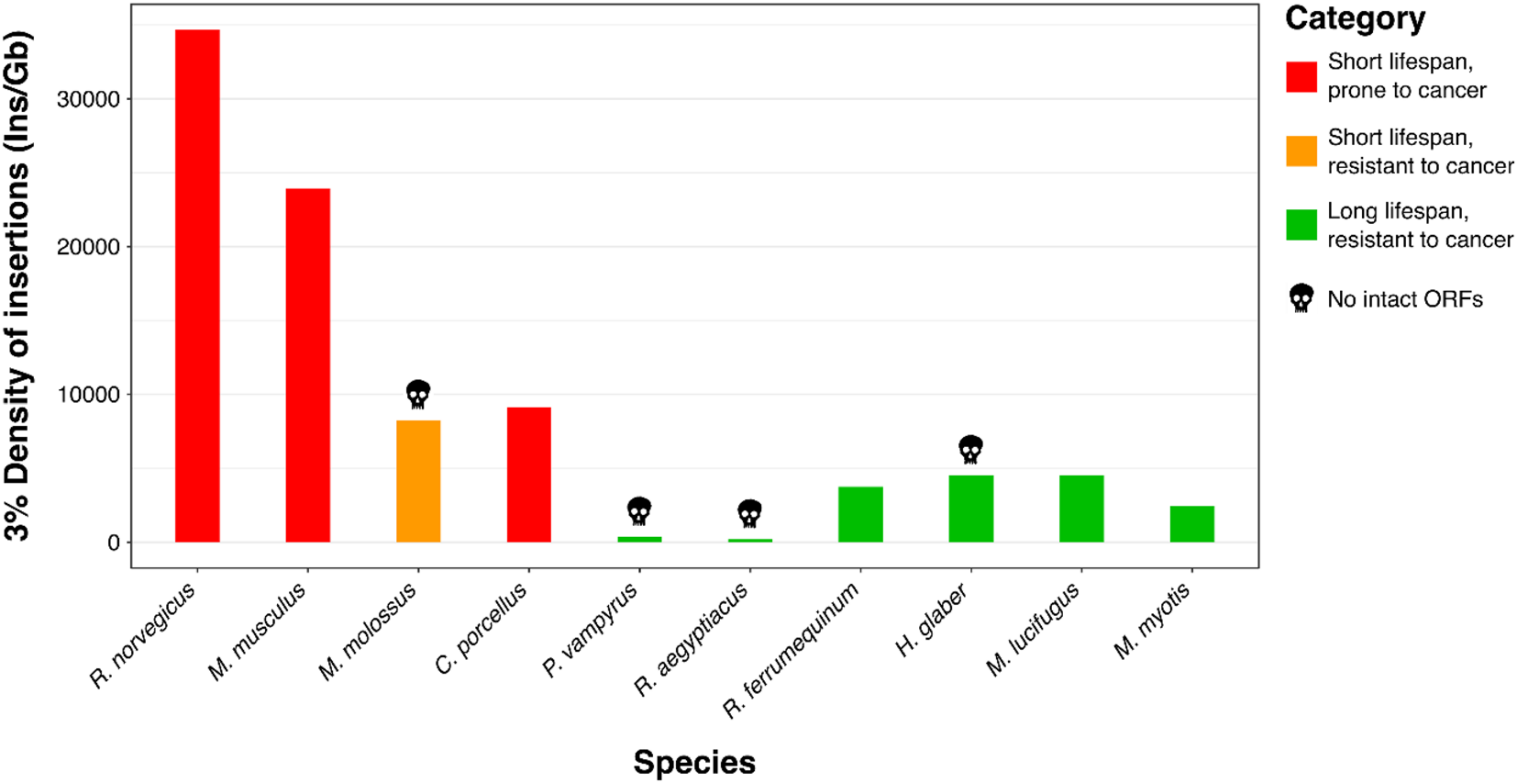
Density of insertion of recent non-LTR retrotransposons. The X-axis shows the species analyzed in increasing order of longevity. The Y-axis shows the density of recent insertions per Gigabase (< 3% divergence from consensus). Red indicates cancer-prone and short-lived species. Orange indicates cancer-resistant and short-lived species. Green indicates cancer-resistant and long-lived species. The black skull indicates the genomes in which no intact LINE open reading frames (ORFs) were detected.

Rodents, in general, show a limited amount of class II transposons with the maximum abundance present in *H. glaber* (2.3%). The genome of *H. glaber* has the largest genome size and its percentage of LINE retrotransposons is relatively high compared to the other rodents (Table 1). Among bats, *M. myotis* and *M. lucifugus* show a marked accumulation of Helitrons (6.93 and 6.18% respectively) in comparison with the other species that show an abundance of Helitrons below 1%. Notably, the short-lived *M. molossus* show the largest genome size and the highest percentage of non-LTR retrotransposons (SINEs and LINEs; Table 2). To investigate how the TE content of rodents and bats changed over time and detect the most recently active transposable elements, we used the RepeatMasker annotation (see “Methods” section) to generate a TE “genomic landscape” for each of the species analysed. The TE landscapes are a visualisation of the proportion of repeats in base pairs (Y-axis) at different levels of divergence (X-axis) calculated as a Kimura 2-p distance^31^ between the insertions annotated and their respective consensus sequences (Figs. 3, 4). The TE landscapes in rodents are mostly dominated by retrotransposons and all of them show an ancestral peak of accumulation between 20 and 30% of divergence (Fig. 3). A small percentage of those ancient TEs is composed of relics of DNA transposons (blue in Fig. 3). The cancer-resistant rodent *H. glaber* show the highest accumulation of retrotransposons dominated by LINEs (green) between 5 and 10% of divergence followed by a dramatic drop corresponding to the most recent history of this genome (Fig. 3). On the other hand, the three cancer-prone rodents accumulated a large number of retrotransposons in recent times. *M. musculus* and *R. norvegicus* share a peak of accumulation between 15 and 20% of divergence, whereas *R. norvegicus* maintains a stable rate of retrotransposition of SINEs. In comparison to bats, rodents do not present high levels of class II transposons (Tables 1 and 2). The TE landscapes of the six bats (Fig. 4) show a higher intra-order diversity in comparison with the four rodents that are homogenous (Fig. 3). In particular, the two megabats belonging to the Pteropodidae family (*Rousettus aegyptiacus* and *Pteropus vampyrus*) presented the lowest TE accumulation (Table 2), whereas *R. aegyptiacus*, *P. vampyrus* and *R. ferrumequinum* have a shared peak of accumulation at 25% of divergence from consensus. The other three species (*M. myotis*, *M. lucifugus* and *M. molossus*), belonging to the clade of Yangochiroptera that evolved around 59 Mya ago^32^, are more heterogeneous at the level of TE accumulation. In fact, the two species belonging the *Myotis* genus share their highest peak of accumulation at 15% of divergence, whereas *M. molossus* has a second more recent taxon-specific peak at 8% of divergence. Despite the two *Myotis* species having the most similar landscapes (likely due to the closer phylogenetic relationship), *M. lucifugus* shows a higher accumulation and diversity of TEs in its recent history (0–3% divergence). On the other hand, *M. myotis* accumulated LTRs with a peak between 4 and 5% of divergence and, in general, the genus *Myotis* has the most pronounced accumulation of DNA transposons and Helitrons. Finally, we can affirm that in all the bat genomes here considered, the non-LTR retrotransposons (LINEs and SINEs) show a “*glaber*-like” dynamic with a decrease of accumulation in correspondence of their most recent evolutionary history (Fig. 4). Since we observed that long-lived species of both orders show little accumulation of non-LTR retrotransposons in recent times, we decided to compare in more detail their activity focusing on elements with a divergence lower than 3% (a proxy for recently inserted TEs) and their density of insertion (DI; Fig. 5).

The density of insertion was calculated as the ratio between the number of non-LTR retrotransposon (SINEs and LINEs) insertions and the assembly size in Gigabase (Fig. 5, Table S1). DI is a parameter used to compare the magnitude of the recent TE accumulation and its potential impact between species with different genome sizes^4^. When applying this measure in rodents, we found that the *H. glaber* showed the lowest DI (4,551) compared to the short-lived and cancer-prone species (Fig. 5). Then, we found lower DIs in bats than in rodents (Fig. 5) except for *M. molossus* (8,269). The lowest DI (< 1000) was observed in *R. aegyptiacus* and *P. vampyrus*. The remaining species *M. myotis*, *M. lucifugus* and *R. ferrumequinum* have a DI ranging between 2000 and 5000. In general, the long-lived bats presented a lower DI compared to the short-lived *M. molossus* (Fig. 5). We also calculated the density of insertion of these retrotransposons per non-overlapping windows (0.5, 1, 1.5 Mb size) across the entire genomes (Figures S1–S3, Table S2). The resulting distribution of TE densities mimic the general DI.

We made a further detailed analysis of LINEs looking for potentially active autonomous non-LTR retrotransposons: we looked for the presence of LINE-related open reading frames (ORFs) with intact protein domains. By doing this, we found that all the analysed rodent genomes present intact LINE ORFs but the genome of *H. glaber* (Fig. 5). In bats, intact LINE ORFs were found only in *M. myotis*, *M. lucifugus* and *R. ferrumequinum* (Fig. 5). Moreover, we found that *M. molossus*, *R aegyptiacus*, *and P. vampyrus* genome have no intact ORFs. Then, we tested if short- and long-lived species statistically differ in terms of density of insertion. We pooled together the four rodents and six bats to apply the Wilcoxon signed rank paired test. Short-lived species were paired with species showing two-fold longer lifespan for a total of 25 comparisons (see “Methods” section). The test resulted in a p-value of 5.96 × 10^–8^ (Table S3).

Finally, we explored the possible correlation between the density of young non-LTR retrotransposons and gene, exon, intron, intergenic region densities but no strong correlation was found (Table S4). Similarly, we tested for the correlation between the TE density and gene density in gene-rich and gene-poor regions, but no general pattern was found (Table S5a–b).

## Discussion

Transposable element activity and accumulation can have manifold effects on genomes and biological phenotypes. Multiple studies have linked TEs to ageing and the development of several diseases including cancer^14,33–36^. Here, we have studied the relationship between TEs and two different aspects of mammal life: longevity and cancer incidence. In rodents, the short lifespan is associated with the presence of cancer^37^. On the other hand, bats are considered cancer-resistant species^38^ (Fig. 1b). The animals considered in this study are known to have different lifespans while sharing similar, small, body sizes (< 2 kg). *H. glaber* is the rodent with the longest lifespan known (31 years) and resistant to cancer while the other rodents show shorter lifespans of 12 (*C. porcellus*), 4 (*M. musculus*) and 3.8 (*R. norvegicus*) years. Chiroptera species, as far as it is currently known, are long-lived species and resistant to cancer (only a few confirmed cases have been reported^39^). Since TEs have been extensively linked to the development of cancer, we investigated the TE content of bats and rodents to see if it is possible to find shared features between long-lived bat and rodent species.

To analyse the TE content of these organisms, we relied on the use of high-quality genome assemblies based on long reads that better represent the actual genomic repetitive content with respect to assemblies based on short reads^1^. The use of long read-based genome assemblies is particularly important when analysing young TE insertions (as in this study) that are highly homogeneous and tend to be underrepresented in short read assemblies^2,40^. On top of that, the combination of long read assemblies and custom TE libraries maximises the representation and annotation of the transposable element content as highlighted here for the genome of *H. glaber* (Fig. 2). The first TE annotation of *H. glaber* without a custom TE library showed a repetitive content of about 25%^41^ but the use of a proper TE library increased the content to ~ 34% (Fig. 2). Finally, the use of long reads increased the total content of *H. glaber* TEs to 38% (Fig. 2, Table 1**)**.

By analysing the TE annotations, we found that the main difference between short- and long-lived species of rodents is represented by a drop in non-LTR retrotransposon accumulation at recent times (0–5% divergence; Fig. 3). Similarly, the long-lived species of bats showed a drop in non-LTR retrotransposon accumulation at recent times (Fig. 4) while presenting an overall accumulation of class II transposons (DNA transposons and Helitrons). Previous studies hypothesised that bats have a higher tolerance for the activity of transposable elements with alternative ways to dampen potential health issues due to this activity^38,42^. Given our observation on the shared drop of non-LTR retrotransposons accumulation in bats and in *H. glaber*, we add to the aforementioned hypothesis, that the specific repression of non-LTR retrotransposon activity may enhance cancer resistance. In fact, the non-LTR retrotransposons are the most prevalent types of TEs in rodents (Fig. 3, Table 1) and the most extensively investigated by biomedical research given that they are the only active TEs in the human genome^43^.

Since the total landscapes of TEs include remote evolutionary dynamics which are unlikely to be associated with the genetics and physiology of cancer and aging, we compared the most recent accumulation of non-LTR retrotransposons between the long-lived and short-lived species considered in this study using the density of recent insertions (0–3% of divergence from consensus). As expected, cancer-prone species present a higher load of recently inserted non-LTR retroelements than cancer-resistant species (Fig. 5). While lifespan of rodents showed a strong relation with the recent activity of non-LTR retrotransposons (Fig. 5), the same type of relation for bats is less clear. However, based on DI comparisons, we noticed that the long-lived bats show a DI similar to the sole long-lived cancer-resistant rodent (*H. glaber*), while the sole short-lived bat (*M. molossus*) shows a DI value more similar to short-lived rodents. Finally, by comparing species with large differences in their lifespans, we reject the hypothesis of independency of non-LTR retrotransposons accumulation with ageing in small mammals (Wilcoxon test, p-value: 5.96 × 10^–8^).

**Figure 6:**
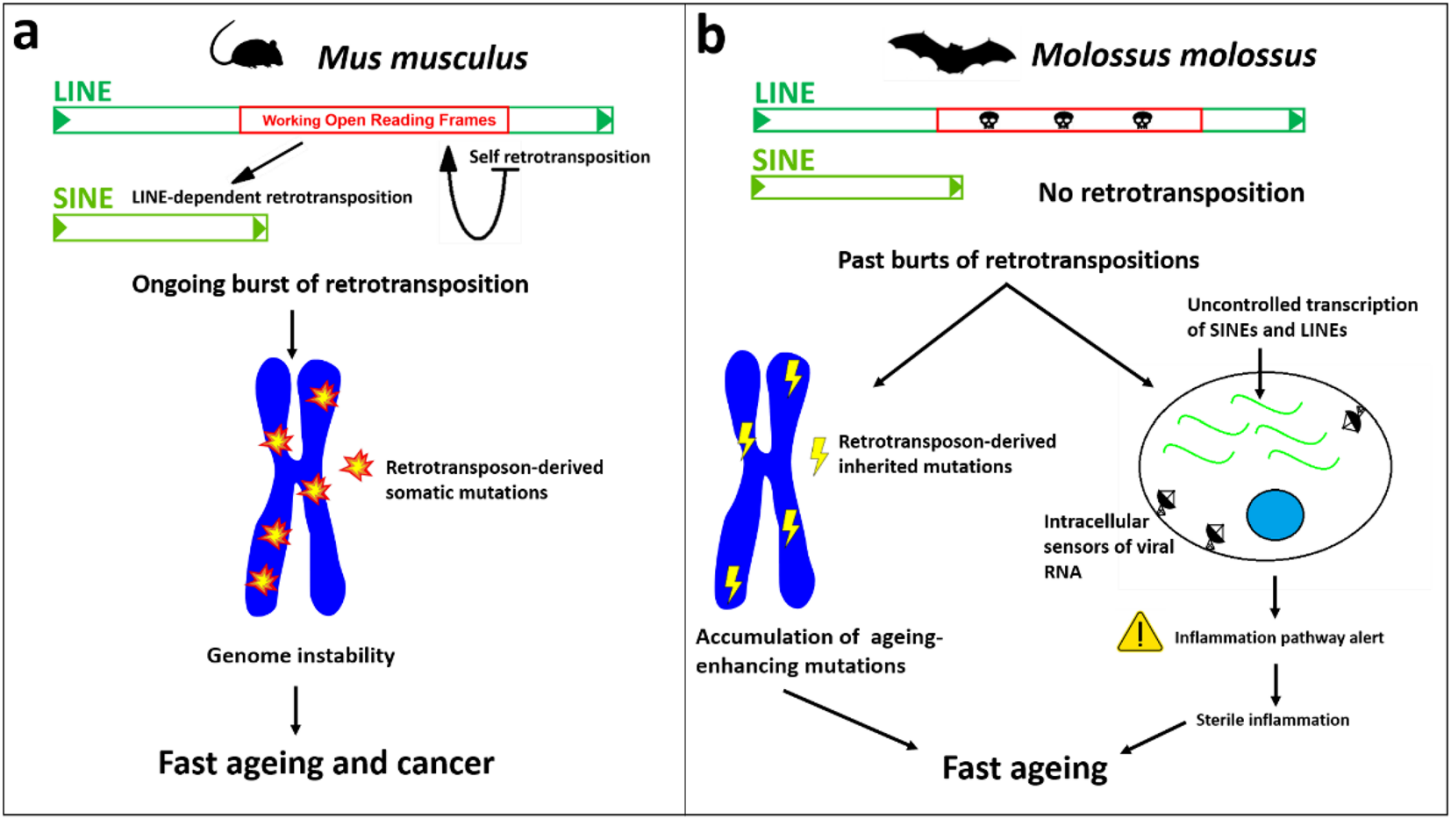
Hypothetical contribution of non-LTR retrotransposons to the fast ageing of rodents and bats. (**a**) *Mus musculus* genome bears active non-LTR retrotransposons that destabilize the genome with continuous events of retrotransposition causing cancer and other diseases that reduce the mouse lifespan. (**b**) *Molossus molossus* is both a cancer-resistant and short-lived species. The high density of SINEs derives from a past accumulation of insertions (Fig. 4), that may have resulted in the accumulation of ageing enhancing mutations, and the continuous transcription of these retroelements may trigger cellular inflammation and cause a widespread sterile inflammation. In this scenario, the fitness of *M. molossus* is not impaired but its lifespan is reduced.

Given the pattern of DI observed in rodents, we speculate that the recent accumulation of non-LTRs may be related to the lifespan of these species (Fig. 1a, 5, 6a). On top of that, we also found that the genome of *H. glaber* does not contain intact ORFs of LINEs, which is another clue for the absence of currently active non-LTR retrotransposons (Fig. 5). The absence of intact LINE ORFs in the long read genome assembly confirms the same observation previously reported on the short read based genome assembly^44^. In bats, we observed that *M. molossus* (shortest lifespan in bats), showed the highest DI, and no intact LINE ORFs (Fig. 5) but has a very high percentage of non-autonomous SINEs compared to long-lived bats (Table 2). SINEs are about 300 bp long and do not present any protein coding sequence, which makes them unable to retrotranspose autonomously: SINEs rely on the exploitation of the retrotransposition machinery provided by LINEs to move throughout the genome (Fig. 6a).

Given that the *M. molossus* genome lacks intact LINE ORFs, it is reasonable to assume that both LINEs and SINEs are currently inactive in this species (Fig. 6b). This begs the question of why *M. molossus* has a short lifespan. The density of recently inserted non-LTR elements in *M. molossus* is 8269 per Gb, which is comparable to the value in *Cavia porcellus* (9,163 insertions per Gb). In contrast, the long-lived rodent *Heterocephalus glaber* has a DI of only 4,550 insertions per Gb, while the long-lived *Myotis lucifugus* has 4,531 insertions per Gb. These findings suggest that future studies on bats should consider two phenomena: first, the potential impact of the accumulation of LINE and SINE insertions on age-related conditions, and second, the cytotoxic effects of an excess of transcribed RNA from these retrotransposons.

A large number of very similar insertions across the chromosomes may cause structural polymorphisms (e.g., through non-allelic homologous recombination events) independently from the retrotransposition itself^45^. For example, in the recent history of the human genome, LINE1-driven ectopic recombination events caused genomic rearrangements that can be responsible for several diseases^46^. Moreover, it has been observed in the African killifishes that an accumulation of detrimental polymorphisms following a burst of TEs can undergo phases of relaxed selection were the mutations eventually promote age-associated diseases and reduced lifespan^47^. Among the killifishes, the turquoise killifish has a lifespan of 4–8 months (likely the shortest lifespan among vertebrates^47^) but despite the accumulation of mutations that leads to a fast ageing, this species is fully adapted to its ecological niche demonstrating that a rapid ageing is not necessarily linked to reduced fitness (Fig. 6b). In addition, SINEs can also exert physiological effects by being transcribed. For example, in humans, SINEs of the *Alu* family have been found to be involved in the inflammatory pathway: upon viral infection, the interferon 1 mediates the overexpression of *Alu* sequences which function as an amplifier of the immune response by stimulating the intracellular viral RNA sensor systems^48^. Despite the involvement of SINEs in the human immune response, the same upregulation of *Alu* elements, or the excessive sensitivity of the viral RNA sensors, has been linked to autoimmune diseases^49^. Furthermore, as non-coding RNAs are emerging as essential components of neuroinflammation, a recent study has included *Alu* SINEs as possible promoters of neurodegenerative diseases^50^. More in general, the increased transcription of non-LTR retrotransposons (both LINEs and SINEs) in humans may contribute to so-called “sterile inflammation” (Fig. 6b), a phenomenon for which a chronic state of inflammation is triggered without the presence of any obvious pathogen and that is exacerbated with age (“inflammaging”)^51^. Therefore, we speculate that the marked accumulation of SINEs in *M. molossus* might trigger phenomena similar to the sterile inflammation and inflammaging that can cause a shortening of its lifespan. For these reasons *M. molossus*, together with the well-known *H. glaber*, may be an exceptional model for biogerontology. Further bioinformatic research can be developed in this respect and may lead to a more precise understanding of the regulation of TEs in the study of longevity and oncology and potential new biomedical applications.

## Methods

### Samples

In order to avoid biases related to potential underestimations of the TE content, in this study we chose those species of mammals from which high-quality genome assemblies were available. The genome assemblies for the 10 species used were retrieved from NCBI and their accession numbers are: *Mus musculus* (GCA_000001635.8) **[**Genome Reference Consortium mouse reference 38], *Rattus norvegicus* (GCA_000001895.4)^52^, *Cavia porcellus* (GCA_000151735.1)^53^, *Heterocephalus glaber* (GCA_014060925.1)^54^, *Rousettus aegyptiacus* (GCA_014176215.1)^55^, *Rhinolophus ferrumequinum* (GCA_014108255.1)^55^, *Molossus molossus* (GCA_014108415.1)^55^, *Pteropus vampyrus* (GCA_000151845.1)^53^, *Myotis myotis* (GCA_014108235.1)^55^, *Myotis lucifugus* (GCA_000147115.1)^53^. The species selected belong to Rodentia and Chiroptera orders. Their most recent common ancestor dates back to the Mesozoic era 90 Mya ago^56^. The large evolutionary distance among these two orders allowed us to find genomic features potentially conserved in all mammals. Body mass is generally positively related to the lifespan^30^ therefore, in our study, we chose to analyse genomes belonging to small-sized mammals (between 12 g and 1.1 kg). The comparisons were carried out using species belonging to the same taxonomical order to maximize their shared evolutionary history and life history traits. These species were also selected and compared based on their lifespans (Fig. 1). The genome assemblies analysed are all based on Sanger or PacBio technologies and selected to maximize their assembled repetitive content^1^. The short^41^ (GCA_000230445.1) and long read-based^54^ genome assemblies of *H. glaber* were compared in order to estimate the differences in TE content due to technological biases.

### Repetitive element libraries and annotation

To analyze the content of transposable elements in genomes, the genome assemblies must be annotated with proper libraries of TE consensus sequences. A consensus sequence represents the reference sequence of a specific subfamily of transposable elements. The repeat annotation software (e.g., RepeatMasker) uses such libraries of consensus sequences to find instances (or insertions) of each repetitive element within the genome assemblies. From the alignments of the TE insertions found in the genome assembly and their consensus sequences, it is possible to estimate the age of such insertions based on the genetic distance given by the alignments: the higher the distance, the higher the age of the insertions. All the species analysed have a species-specific repeat library already available except for *H*. *glaber*. Therefore, we made a de novo repeat library of *H. glaber* using RepeatModeler2 with the option -LTRstruct^57^. The resulting consensus sequences were merged with the ancient mammalian TE families (LINE2 and MIR SINEs) downloaded from RepBase (https://www.girinst.org/repbase). All the genome assemblies of rodents (but *H. glaber*) were masked using RepeatMasker (v. 4.1.0)^58^ and the Rodentia-specific library (Repbase release 20181026). Similarly, the bat genome assemblies were masked using the Chiroptera-specific library from Repbase merged with the curated TE libraries produced by Jebb et al.^55^. The RepeatMasker annotations were performed using the options -a -xsmall -gccalc -excln. The annotations were then visualized as “genomic landscapes”: barplots that show in the X-axis the genetic distances from the consensus sequences (calculated as Kimura 2-p distance^31^) and in Y-axis the percentage of the genome occupied by each TE category (Figs. 3, 4). The barplots were made using the ggplot2 R package. For each species, the genome assembly size and the percentage of each TE categories (Tables 1, 2, Fig. 2) were retrieved from the table files produced by RepeatMasker.

### Density of young non-LTR retrotransposons

To evaluate the recent activity of non-LTR retrotransposons (LINEs and SINEs), we selected all the non-LTR retrotransposon hits found by RepeatMasker with a divergence from consensus lower than 3%. The 3% threshold is a proxy for most recently inserted elements^3,4^. To minimise the overestimation of insertions due to the fragmentation of the repeat annotation, we controlled for fragments belonging to the same insertion as RepeatMasker assign them the same identifier. Then we calculated the density of insertion (DI) for each species as the ratio between the number of recent insertions and the corresponding assembly size expressed in Gigabase:

$$Density\; of\; insertion\; \left( {DI} \right) = \frac{Number\; of \;insertions}{{Assembly \;size\; \left( {Gb} \right)}}$$

We expect that the long-lived species are associated with lower DI with respect to lower lived species. To test this hypothesis, we pooled together bats and rodents creating all possible comparisons of long-lived with short-lived species. Each pair of species were chosen as follows: long-lived species lifespan must be at least two-fold longer than the second species’ lifespan; for example: *H. glaber* (31 years) versus *M. molossus* (5.6 years). A total of 25 pairs of species were then compared (Table S3). To test this association, we chose Wilcoxon signed rank paired test (one tailed; alpha: 0.05) since the data are not normally distributed.

We also calculated the density of the number of young non-LTR retrotransposon insertions across the genomes in non-overlapping windows of 0.5, 1 and 1.5 Mb, to then calculate the mean density and standard deviation.

### Density of young non-LTR retrotransposons and their correlation to genomic features

To understand if the densities of insertions found in these genomes correlate with the density of genes, exons, introns, and intergenic regions, we collected all the gene annotations available for the species of this study. We found available on NCBI the gene annotations for all the genome assemblies except for *Heterocephalus glaber*, *Pteropus vampyrus *and *Myotis lucifugus*. We then calculated the density of these genomic features for non-overlapping windows of 0.5, 1 and 1.5 Mb as the number of features per unit of window size. To test the correlation between the TE density and the other genomic features, we used the Spearman rank correlation test.

In addition, we tested if there was a correlation between the TE density and gene density in gene-rich and gene-poor regions (windows) specifically. We categorized gene-rich and gene-poor windows based on their gene density distribution. The windows showing a gene density value less or equal to the 0.25 percentile of the distribution were categorized as gene poor. The windows with a gene density value greater or equal to the 0.75 percentile of the gene density distribution were categorized as gene rich. The correlation between the TE and gene densities were tested with the Spearman rank correlation test. Finally, we tested if the TE density in gene-rich and gene-poor regions (windows) statistically differed by using the Wilcoxon rank sum test.

### Intact ORFs detection

To check the presence of intact LINE retrotransposons (therefore potentially active), we looked for complete open reading frames (ORFs) that encode for the enzymatic machinery used by LINEs to retrotranspose themselves and the non-autonomous SINE elements. In each species the sequences of LINEs were obtained with BEDTools getfasta command^59^ using the LINE coordinates annotated by RepeatMasker. A fasta file with the LINE sequences for each species was produced and used as input for the R script orfCheker.R (https://github.com/jamesdgalbraith/OrthologueRegions/blob/master/orfChecker.R) to find intact LINE ORFs. The script considers a LINE ORF to be intact if it contains both complete reverse transcriptase and endonuclease domains.

## Supplementary Information

Supplementary Information 1.

Supplementary Information 2.

Supplementary Information 3.

Supplementary Information 4.

Supplementary Information 5.

Supplementary Information 6.

Supplementary Information 7.

Supplementary Information 8.

Supplementary Information 9.

Supplementary Information 10.

Supplementary Information 11.

Supplementary Information 12.

Supplementary Information 13.

Supplementary Information 14.

## Data Availability

All the genome assemblies used in this study were retrieved from NCBI: *Mus musculus*: GCA_000001635.8; https://www.ncbi.nlm.nih.gov/data-hub/genome/GCA_000001635.8/. *Rattus norvegicus*: GCA_000001895.4; https://www.ncbi.nlm.nih.gov/data-hub/genome/GCF_000001895.5/. *Cavia porcellus*: GCA_000151735.1; https://www.ncbi.nlm.nih.gov/data-hub/genome/GCF_000151735.1/. *Heterocephalus glaber* (based on long reads): GCA_014060925.1; https://www.ncbi.nlm.nih.gov/data-hub/genome/GCA_014060925.1/. *Heterocephalus glaber* (based on short reads): GCA_000230445.1; https://www.ncbi.nlm.nih.gov/data-hub/genome/GCF_000230445.1/. *Rousettus aegyptiacus*: GCA_014176215.1; https://www.ncbi.nlm.nih.gov/data-hub/genome/GCF_014176215.1/. *Rhinolophus ferrumequinum*: GCA_014108255.1; https://www.ncbi.nlm.nih.gov/data-hub/genome/GCA_014108255.1/. *Molossus molossus*: GCA_014108415.1; https://www.ncbi.nlm.nih.gov/data-hub/genome/GCF_014108415.1/. *Pteropus vampyrus*: GCA_000151845.1; https://www.ncbi.nlm.nih.gov/data-hub/genome/GCF_000151845.1/. *Myotis myotis*: GCA_014108235.1; https://www.ncbi.nlm.nih.gov/data-hub/genome/GCF_014108235.1/. *Myotis lucifugus*: GCA_000147115.1; https://www.ncbi.nlm.nih.gov/data-hub/genome/GCF_000147115.1/. The code used for the analysis can be found at: https://github.com/marcoricci20/TEanalysis/.
