# Supplementary material for: Comparative analysis of bats and rodents’ genomes suggests a relation between non-LTR retrotransposons, cancer incidence, and ageing
Source: Sci Rep. 2023 Jun 3;13:9039. doi: 10.1038/s41598-023-36006-6 (PMC10239488; doi:10.1038/s41598-023-36006-6)
Supplement: Supplementary file 13 — Supplementary Information 13. [file 41598_2023_36006_MOESM13_ESM.docx]

# Supplementary Figures


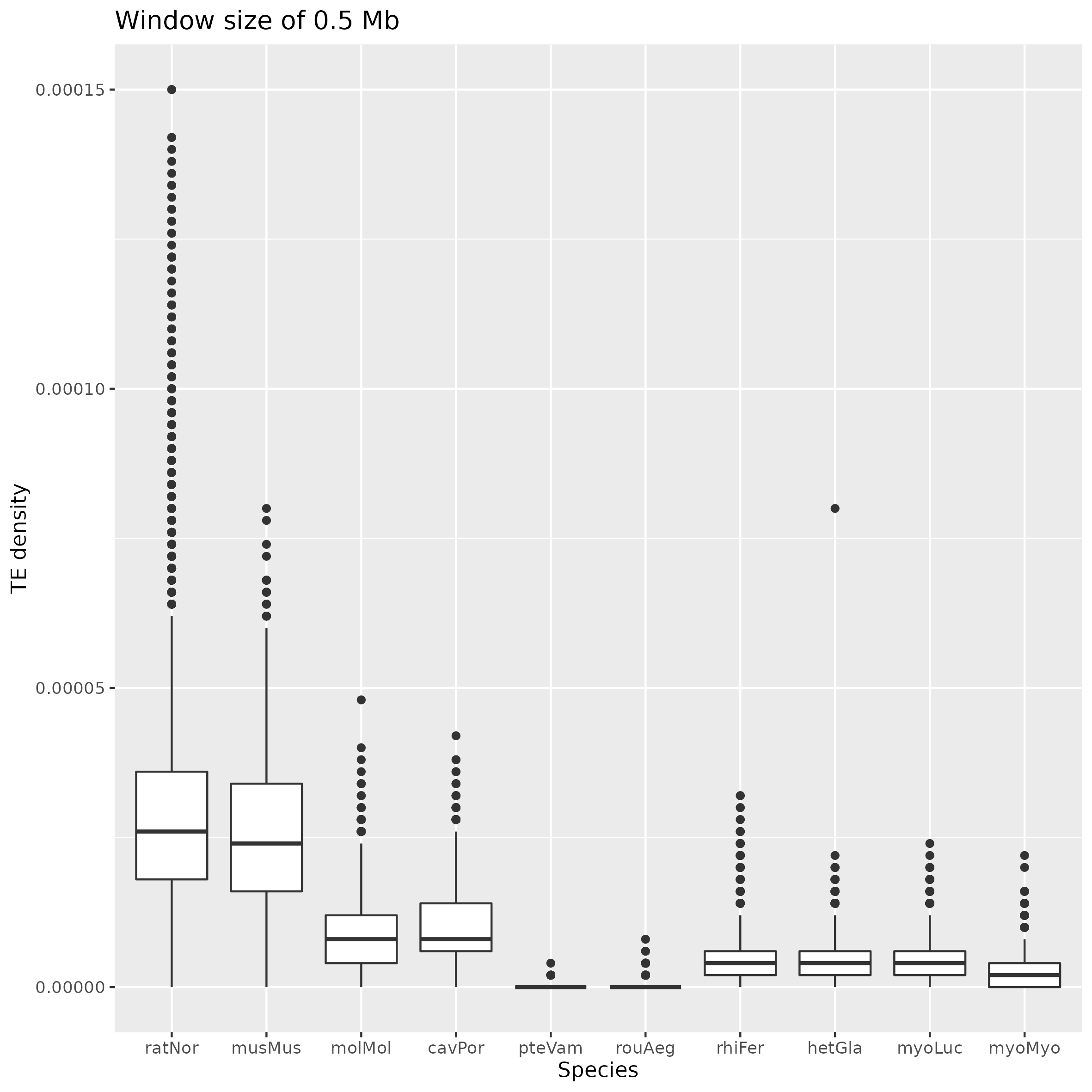


**Figure S1**. Boxplots of the distribution of the density of non-LTR retrotransposons with divergence less than 3% in windows of 0.5 Mb.


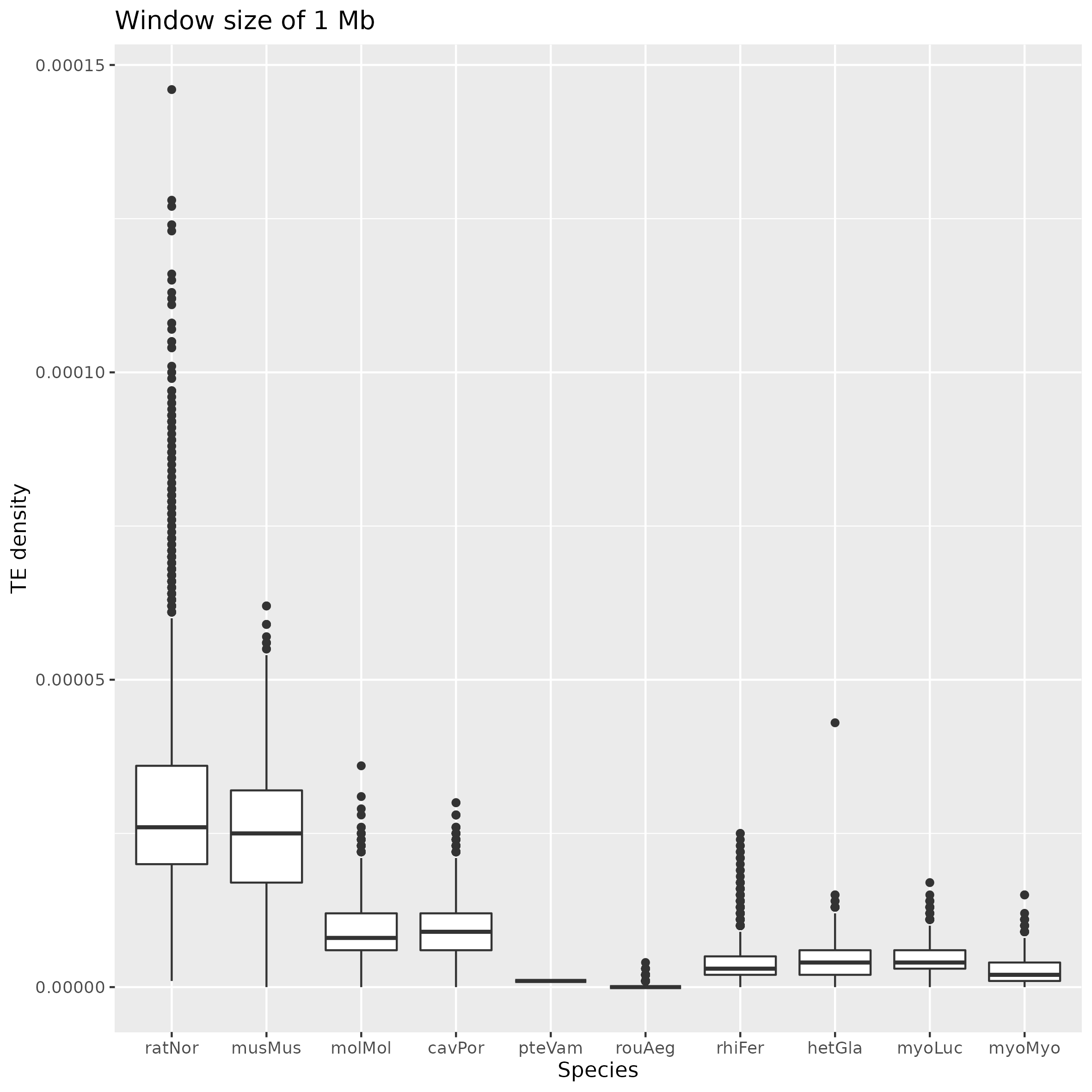


**Figure S2.** Boxplots of the distribution of the density of non-LTR retrotransposons with divergence less than 3% in windows of 1 Mb.


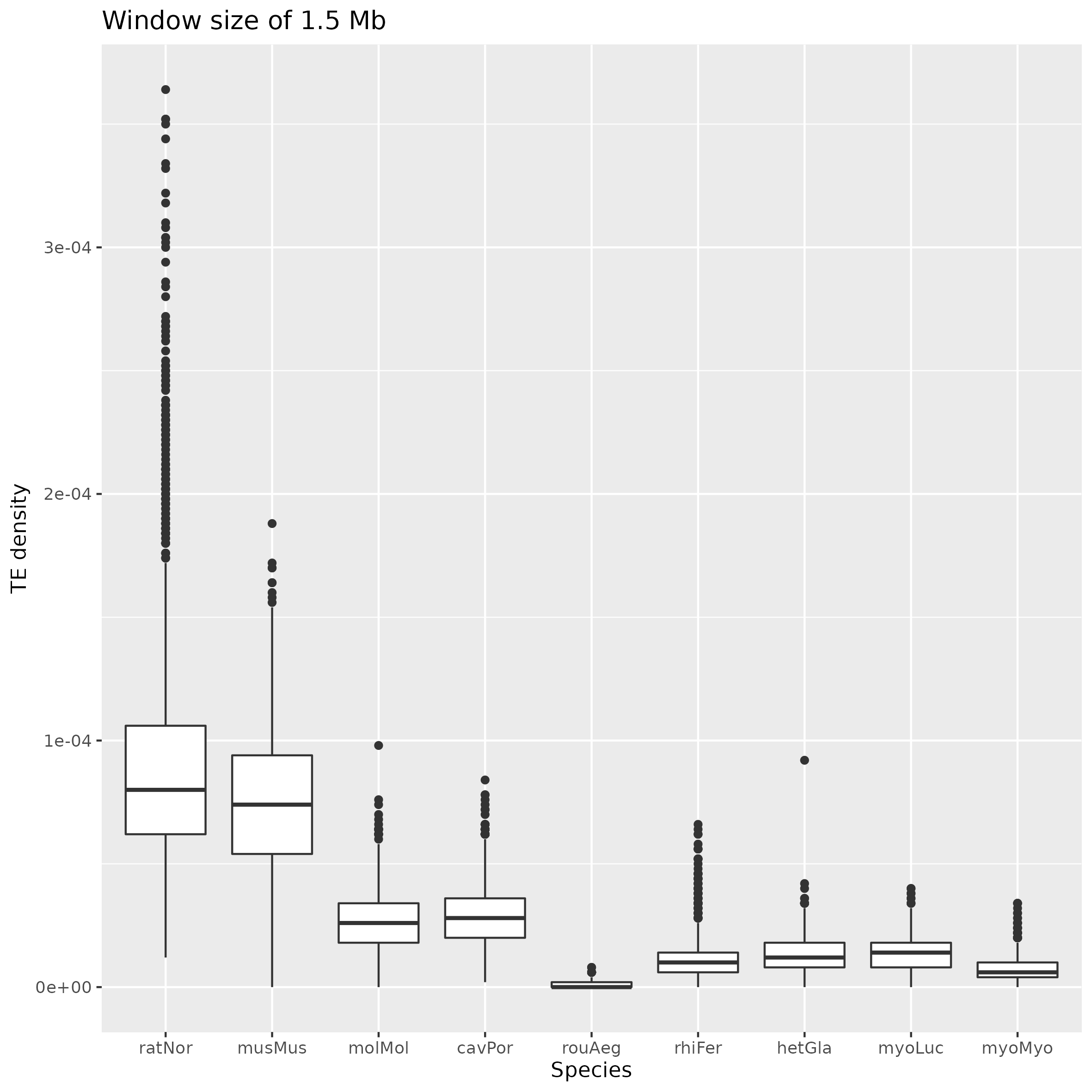


**Figure S3**. Boxplots of the distribution of the density of non-LTR retrotransposons with divergence less than 3% in windows of 1.5 Mb.


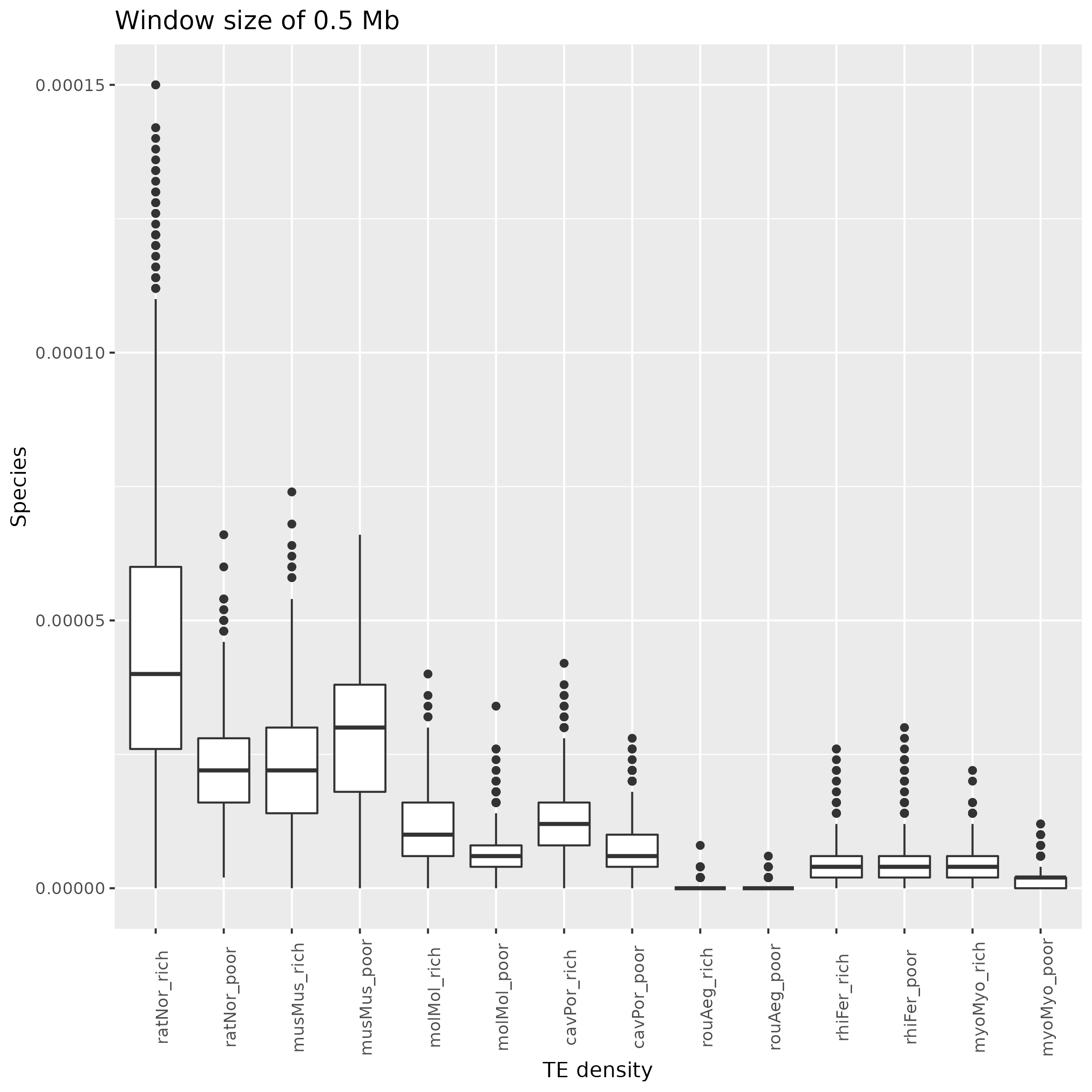


**Figure S4**. Boxplot of the distribution of TE densities in gene-rich and gene-poor regions calculated over a window size of 0.5 Mb.


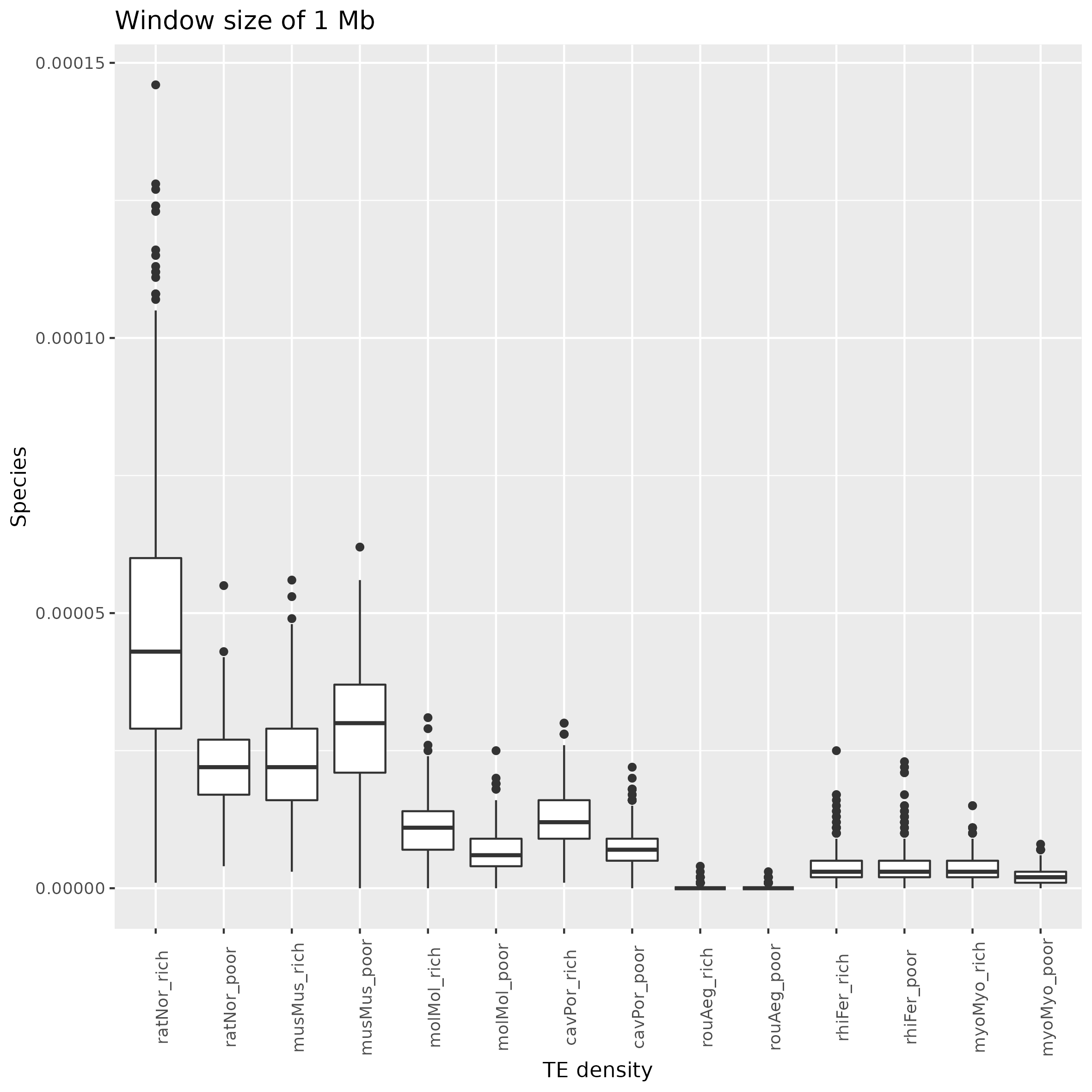


**Figure S5**. Boxplot of the distribution of TE densities in gene-rich and gene-poor regions calculated over a window size of 1 Mb.


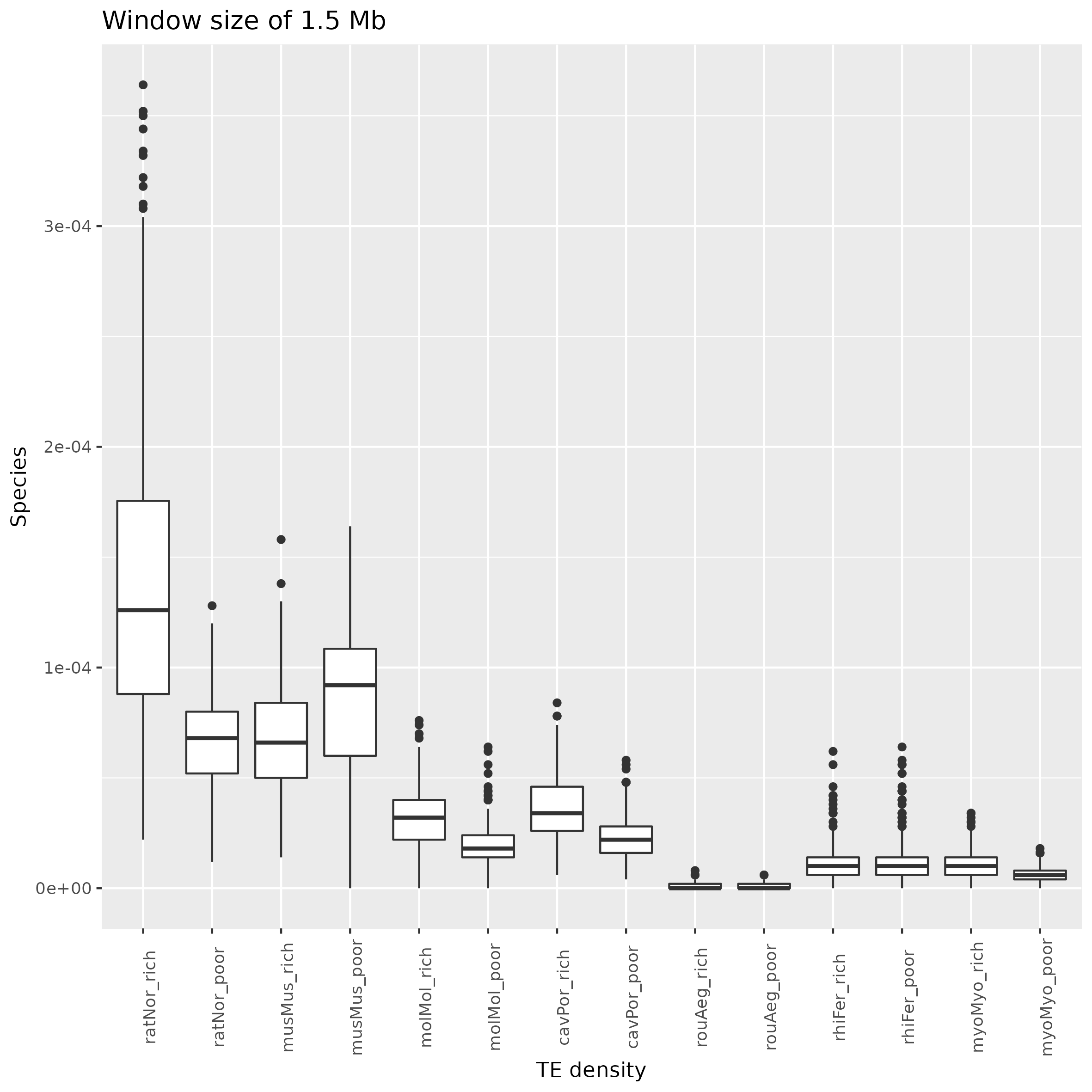


**Figure S6**. Boxplot of the distribution of TE densities in gene-rich and gene-poor regions calculated over a window size of 1.5 Mb.
